# The Use of Deep Learning in Distinguishing Chalazion and Eyelid Mass

**DOI:** 10.1155/joph/8878251

**Published:** 2026-04-17

**Authors:** Anfei Li, Gabriella Schmuter, Shehroz Rana, Sayyada Hyder, Pratik Patel, Amy Hembree, Amylisa Philips, Simone Prather, Mark Curato, Kira Segal, Kyle J. Godfrey, Gary Lelli

**Affiliations:** ^1^ Department of Ophthalmology, Weill Cornell Medicine, New York City, New York, USA, cornell.edu; ^2^ Department of Emergency Medicine, Weill Cornell Medicine, New York City, New York, USA, cornell.edu; ^3^ Department of Medicine, Weill Cornell Medicine, New York City, New York, USA, cornell.edu; ^4^ Department of Neurological Surgery, Weill Cornell Medicine, New York City, New York, USA, cornell.edu

**Keywords:** AI, chalazion, eyelid mass

## Abstract

**Purpose:**

Our study investigated the ability of artificial intelligence to differentiate eyelid lesions to support its potential use as a tool to better inform referrals to oculoplastic surgery specialists by other healthcare providers. Specifically, our study tested artificial intelligence’s ability to distinguish benign chalazia from alternative eyelid masses that may require advanced subspecialized care with oculoplastic specialists.

**Methods:**

This retrospective case–control study included 206 photographs of diagnosed chalazia from 183 patients and 517 photographs from 486 patients with non‐chalazia eyelid lesions to train and test a convolutional neural network (CNN). Network architectures including VGG‐16, VGG‐19, ResNet50, Xception, and MobileNetV2 were trained. Their performances were compared using the area under the curve (AUC) as the main outcome metric. Additionally, performances of CNN models were compared to those of frontline physicians.

**Results:**

VGG‐16 and VGG‐19 architectures achieved meaningful performance when trained with photographs of chalazion and eyelid mass achieving AUCs of 0.797 and 0.703, respectively. Adjusting detection thresholding allowed the VGG‐16 and VGG‐19 models to achieve sensitivity of 93% and 98% in predicting eyelid mass, respectively. This was an improvement over classification by frontline physicians who achieved an accuracy of 61% and a sensitivity of 65% for mass detection.

**Conclusions:**

We showed that using a CNN trained with clinical external photographs could successfully distinguish a chalazion from an alternative eyelid mass, supporting its potential use as a tool for healthcare providers to assist in determining whether a mass requires oculoplastic referral for subspecialty care.

## 1. Introduction

The utility of artificial intelligence (AI) has progressively expanded over the past decade in health care, including ophthalmology [1, 2]. The modern‐day ophthalmic evaluation augments the physical examination with myriad technologies amenable to improvement with AI. Examples within ophthalmology demonstrating effective utilization of AI include evaluations of diabetic retinopathy, age‐related macular degeneration, retinopathy of prematurity, glaucoma [1], thyroid eye disease [3], and orbital masses [4]. Eyelid masses and lesions are a common health concern for which patients typically seek care with optometrists, primary care physicians, emergency physicians, and general ophthalmologists. These eyelid changes, ranging from benign inflammation to early malignancy, may appear clinically similar on inspection or examination, especially to those with less ophthalmic and oculoplastic training. Identification of eyelid lesions is of particular importance as biopsy may be needed to evaluate the malignant potential of the ambiguous masses. Various technologies, such as noninvasive meibography, have been studied to assist with the accurate characterization of eyelid masses [5]. Nevertheless, it remains clinically difficult to accurately diagnose eyelid masses, with some masses, such as sebaceous gland carcinoma, carrying high malignant potentials [6, 7]. An improvement in the initial clinical diagnosis and characterization of these lesions could reduce morbidity and mortality associated with eyelid malignancies, optimize utilization of medical resources by reducing unnecessary eyelid biopsies, and enhance the quality and accessibility of patient care. With the above goal in mind, studies from Asia have successfully demonstrated AI’s ability to distinguish between benign and malignant masses, but chalazia, an infectious/inflammatory process distinct from periocular masses and readily treatable by frontline providers, were not specifically distinguished. Additionally, patient ethnicity and skin color were limited due to the uniformity of the Asian populations [8, 9]. There are no studies at this point in time, to the best of the authors’ knowledge, implicating the utilization of publicly available AI models in differentiating a chalazion from an alternative eyelid mass using a real‐world dataset where photographs were obtained in real clinical encounters and skin complexions varied greatly. Successful use of AI on such heterogeneous real‐world clinical photograph for lesion identification has been documented in dermatological literature but has yet to be demonstrated in eyelid lesions. In this work, we trained and tested AI’s ability to correctly differentiate chalazion from eyelid mass based on real‐world clinical external photographs from an ethnically diverse patient population, as a proof‐of‐concept study demonstrating the capability of AI models to function as a screening tool to assist frontline providers, not specifically trained in eyelid lesion differentiation, and identify self‐limited chalazion from potentially malignant masses.

## 2. Methods

### 2.1. Study Population and Data Acquisition

A total of 10,000 patients with CPT code of 67,840 (excision of eyelid lesion) or ICD code of H00.^∗^ (chalazion and hordeolum, ^∗^ for wildcard; Supporting Tables 1 and 2 for full list of ICD codes and diagnoses) seen at a single academic institution between 2005 and 2020 were randomly selected by a repository database and reviewed. All patients with clinical photograph(s) of the eyelid lesion(s) and a confirmed diagnosis of either a malignant eyelid lesion, benign eyelid lesion, or chalazion were included in the study. Due to the photograph requirement, all patients before 2010 were excluded due to the lack of photograph availability in the medical record. Diagnoses of malignant or benign lesions were based on the pathology result of the biopsy as interpreted by the department of pathology. Diagnosis of a chalazion was based on either the result of the biopsy or clinical diagnosis with resolution on follow‐up after treatment. A total of 669 patients with 723 eyes were included in the study. One hundred and eighty‐three patients with 206 eyes had been diagnosed to have a chalazion, and 486 patients with 517 eyes had been diagnosed to have an alternative eyelid mass (either malignant or benign). Race information was collected based on standard reporting of white, black, Asian, other, or declined. A detailed distribution of demographic information can be found in Table 1. All photographs were taken using an iPhone or Android available at the time of the visit with direct access to electronic medical records. There was no specific software, room location, or lighting equipment used for photograph acquisition as this is a retrospective study using photographs from real clinical encounters. There was no specific requirement for angle, lighting, resolution of the photographs, or eyelid position (open or closed; Figure 1) as long as the photograph demonstrated the lesion of interest. Data collection and study design were approved by the Weill Cornell Medicine Institutional Review Board. The study design complies with the Declaration of Helsinki.

**TABLE 1:** Demographic distributions.

	**Total**	**Chalazion**	**Mass**	**p** **value**

Sample size	669 patients	183 patients	486 patients	—
	723 eyes	206 eyes	517 eyes	
Mean age	56.4	49.2	59.1	*p* < 0.001
Gender	400F/269M	102F/81M	298F/188M	*p* = 0.190
Race				
White	359	89	270	*p* = 0.284
Black	38	9	29	
Asian	28	6	22	
Other	74	24	50^∗^	
Declined	170	55	115	
Open/close	604/119	175/31	429/88	*p* = 0.518

^∗^Indicates one Native American and Alaskan.

FIGURE 1Example images of (a) chalazion with eye open (above) and closed (below). (b) Benign lesion with eye open (above) and closed (below). (c) Malignant lesion with eye open (above) and closed (below).

**Figure:**
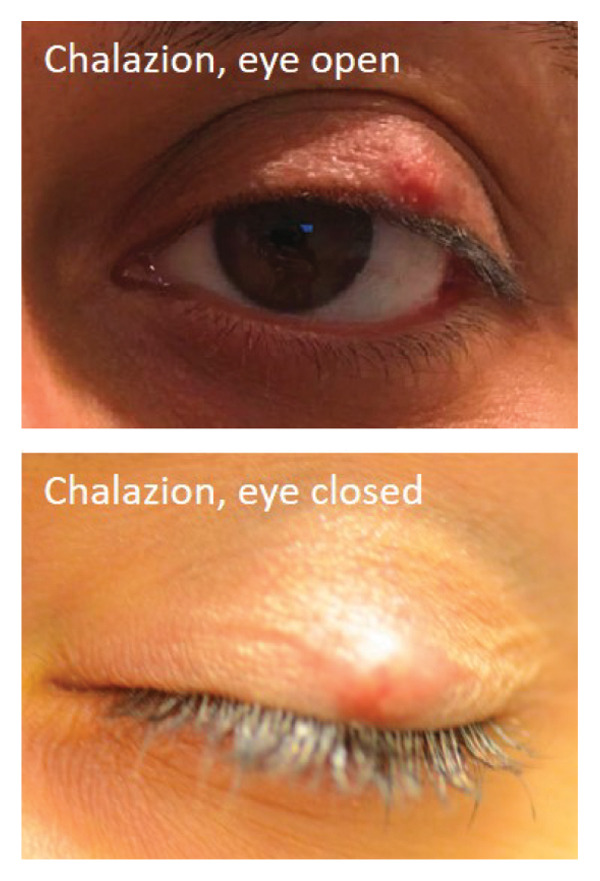
(a)

**Figure:**
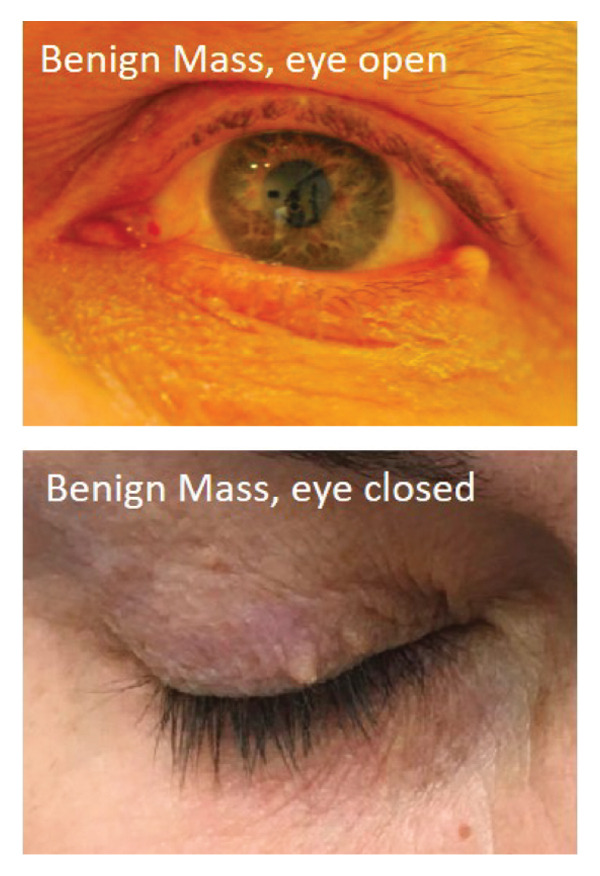
(b)

**Figure:**
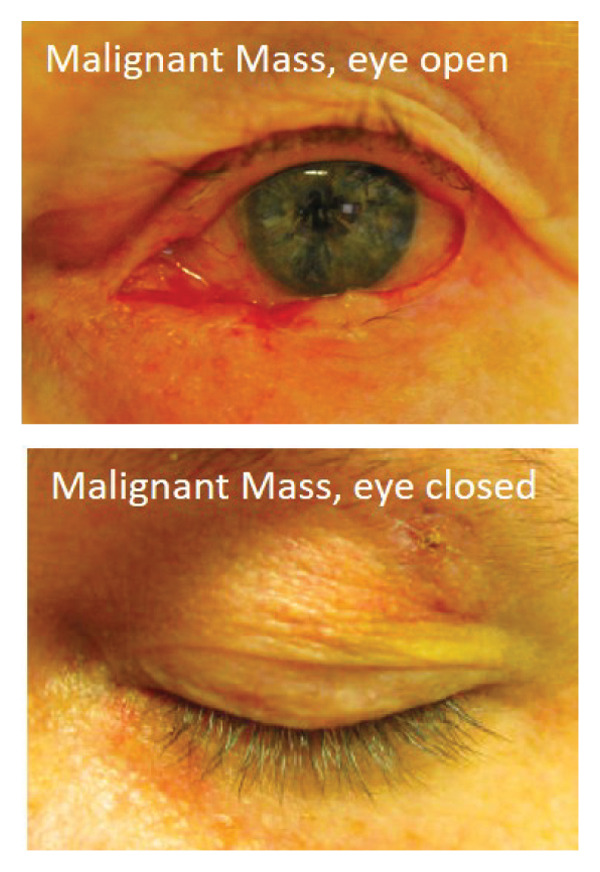
(c)

### 2.2. Image Processing and Data Split

Clinical images were cropped to include the lesion and the entire eye including both upper and lower eyelids. Representative images are shown in Figure 1. Images were standardized to 224 × 224 or 299 × 299 pixels depending on the standard input size of the model used. No additional manual labeling or segmentation was applied to the standardized images. The standardized images were split into training, validation, and testing sets in an 8:1:1 ratio. The training dataset was augmented with horizontal and vertical flips to increase variability for the training. Due to imbalance in the input dataset (more eyelid lesions than chalazia), the input weight of each class was calculated for a weighted modeling. In the weighted modeling, the influence of each photograph on the model is determined by the relative abundance of its class. As there are roughly 2.5 times more mass photographs than chalazion photographs, each chalazion photograph will influence the model 2.5 times more than each mass photograph to counterbalance the relative abundance of mass photographs in the training dataset.

### 2.3. Convolutional Neural Network (CNN)

Keras package (Version 2.3.1, ONEIROS) in Python (Version 3.7.6) was used for this analysis. Pretrained Xception, Visual Geometry Group (VGG)‐16, VGG‐19, ResNet50, and MobileNetV2 models were loaded from Keras package as base model. The descriptions of the pretrained models can be found on the official Keras website (https://keras.io/api/applications/). Four additional dense layers (three relu activation and one sigmoid activation) and two additional dropout layers (0.5 dropout rate) were added to the pretrained models for retraining. Stochastic gradient descent (SGD) optimizer, batch size of 16, and epoch of 100 were used for training parameters. Using the training datasets described above, the model was fitted using weighted training to distinguish an eyelid mass from a chalazion. Trained models were tested using the previously unseen testing set. Gradient‐weighted class activation mapping (Grad‐CAM) from Keras package (https://keras.io/examples/vision/grad_cam/) was used for feature visualization.

### 2.4. Physician Classifications

One emergency medicine attending physician, one optometry attending physician, two emergency medicine residents, one primary care resident, and one oculoplastic fellow from Weill Cornell Medicine participated in the classification of the photographs in the testing set used for model testing. Classifying physicians were instructed to label each photograph as chalazion or mass independently. Performances were calculated individually and as a group. There was no protected/personal health information in the photographs provided.

### 2.5. Statistical Analysis

The area under the curve (AUC) calculation was done using the auc() function from the sklearn package in Python. Demographic data were compared between eyelid mass and chalazion groups using two‐sided Student’s *t*‐test or Fisher’s exact test.

## 3. Results

Of the 699 patients (723 eyes), 183 patients (206 eyes) had a diagnosis of a chalazion and 486 patients (517 eyes) had a diagnosis of an eyelid mass (whether malignant or benign). The average age of the eyelid mass group was 59.1, which was significantly higher than 49.1 for the chalazion group (*p* < 0.001). In both groups, there were more female patients than male patients, but there was no statistical difference in gender distribution between the two groups (*p* = 0.190). Race distribution was also similar between the two groups with white being the predominant race for both groups (*p* = 0.284). Both groups contained photographs of mostly opened eyes and a small portion of closed eyes, and there was no statistical difference in proportion between the two groups (*p* = 0.518, Table 1).

After 100 epochs of training with pretrained models, the area under the curve (AUC) for each model was calculated from the testing dataset. The VGG‐16 and VGG‐19 models showed a significantly improved performance relative to chance with AUCs of 0.797 and 0.703, respectively. However, Xception, ResNet50, and MobileNetV2 had limited performance with AUCs of 0.425, 0.468, and 0.454, respectively (Figure 2). Training was extended to 150 and 200 epochs but no significant improvement in performance was observed, as most models reached performance plateau between 50 and 80 epochs (data not shown).

FIGURE 2Performance of various CNN models. (a) Area under the precision–recall curve. The area under the precision–recall curve was 0.425 for Xception model, 0.468 for ResNet50 model, 0.703 for VGG‐19 model, 0.797 for VGG‐16 model, and 0.454 for MobileNetV2 model. (b) Precision–recall curve of the tested models. No skill line (guess) is denoted with dashed black line.

**Figure:**
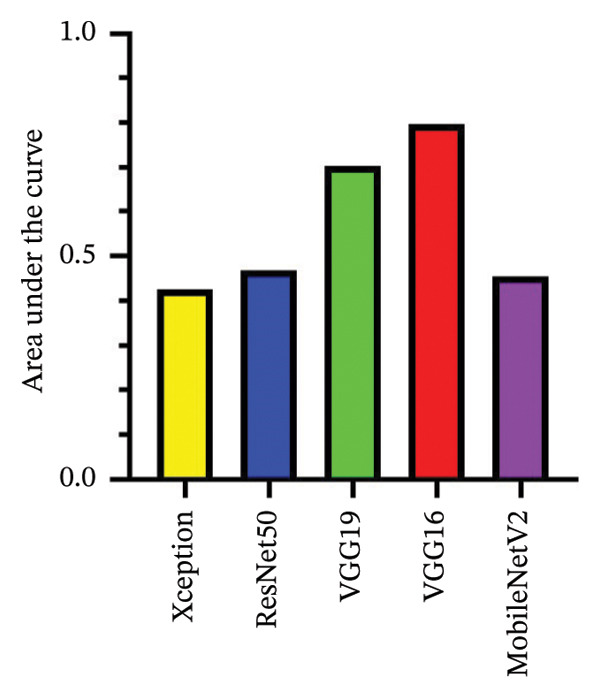
(a)

**Figure:**
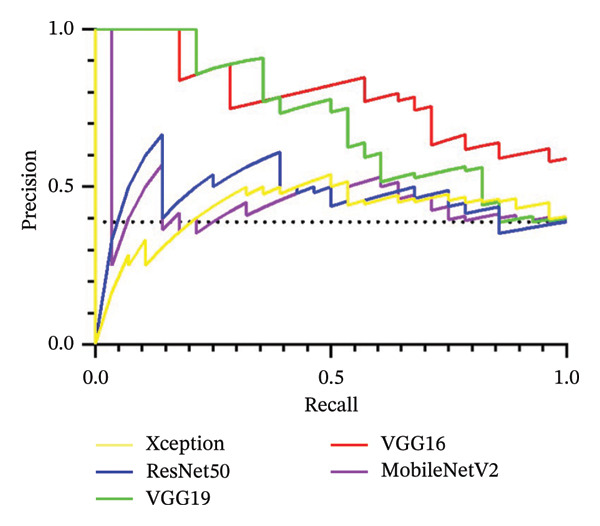
(b)

Consistent with AUCs, VGG models also demonstrated improved performance when looking at accuracy in predicting the testing dataset with VGG‐16 achieving 76% accuracy (loss of 0.57) and VGG‐19 achieving 63% accuracy (loss of 0.64) when the prediction threshold is set to 0.5 (i.e., model will predict chalazion when the model is more than 50% confident). Specifically, both models were proficient in correctly identifying chalazion, but had false negatives when predicting an eyelid mass. With the same threshold, Xception, ResNet50, and MobileNetV2 achieved relatively lower performance—52% (loss of 0.84), 44% (loss of 0.85), and 39% (loss of 0.80), respectively—due to predicting most lesions as chalazion (Table 2). There was no performance difference among subgroups, although the testing set was relatively small for some subgroups. Specifically, subgroup accuracies for VGG‐16 were 76% (26/34) for Whites, 80% (4/5) for Blacks, 80% (4/5) for Asians, 70% (7/10) for others, and 78% (14/18) for patients who declined to answer. When the prediction threshold was increased to 0.6 and 0.7, VGG models maintained relatively high accuracies in the 70%–80% range, but demonstrated higher sensitivity toward mass while also suffering higher false positives (Table 3). Features used by the VGG‐16 model were visualized using Grad‐CAM function (Supporting Figure 1), demonstrating detection of perilesional features.

**TABLE 2:** Performance confusion matrix based on threshold of > 0.5 for chalazion.

**Threshold 0.5**	**Xception prediction**			**VGG-19 prediction**			**VGG-16 prediction**			**ResNet50 prediction**			**MobileNetV2 prediction**		
	**Mass**	**Chal.**		**Mass**	**Chal.**		**Mass**	**Chal.**		**Mass**	**Chal.**		**Mass**	**Chal.**	

True mass	10	34	TPR 0.23	22	22	TPR 0.5	28	16	TPR 0.64	7	37	TPR 0.16	0	44	TPR 0.00
True chalazion	1	27	FPR 0.04	5	23	FPR 0.18	1	27	FPR 0.04	3	25	FPR 0.11	0	28	FPR 0.00
	PPV 0.91	NPV 0.44	ACC 0.51	PPV 0.81	NPV 0.51	ACC 0.63	PPV 0.97	NPV 0.63	ACC 0.76	PPV 0.70	NPV 0.40	ACC 0.44	PPV N/A	NPV 0.39	ACC 0.39

*Note:* ACC = accuracy. PPV, positive predictive value (also known as precision); TRP, true positive rate (also known as recall or sensitivity).

Abbreviations: FPR, false‐positive rate; NPV, negative predictive value.

**Table 3:** Performance confusion matrix based on threshold of > 0.6 and > 0.7 for chalazion.

**Threshold 0.6**	**Xception prediction**			**VGG-19 prediction**			**VGG-16 prediction**			**ResNet50 prediction**			**MobileNetV2 prediction**		
	**Mass**	**Chal.**		**Mass**	**Chal.**		**Mass**	**Chal.**		**Mass**	**Chal.**		**Mass**	**Chal.**	

True mass	11	33	TPR 0.25	40	4	TPR 0.91	31	13	TPR 0.70	9	35	TPR 0.20	0	44	TPR 0.00
True chalazion	6	22	FPR 0.21	13	15	FPR 0.46	2	26	FPR 0.07	3	25	FPR 0.10	0	28	FPR 0.00
	PPV 0.65	NPV 0.40	ACC 0.46	PPV 0.75	NPV 0.79	ACC 0.76	PPV 0.94	NPV 0.66	ACC 0.79	PPV 0.75	NPV 0.42	ACC 0.47	PPV N/A	NPV 0.39	ACC 0.39

**Threshold 0.7**	**VGG-16 prediction**			**Xception prediction**			**VGG-19 prediction**			**ResNet50 prediction**			**MobileNetV2 prediction**		
	**Mass**	**Chal.**		**Mass**	**Chal.**		**Mass**	**Chal.**		**Mass**	**Chal.**		**Mass**	**Chal.**	

True mass	27	17	TPR 0.61	43	1	TPR 0.98	41	3	TPR 0.93	44	0	TPR 1.00	44	0	TPR 1.00
True chalazion	13	15	FPR 0.46	20	8	FPR 0.71	10	18	FPR 0.36	28	0	FPR 1.00	28	0	FPR 1.00
	PPV 0.68	NPV 0.47	ACC 0.58	PPV 0.68	NPV 0.89	ACC 0.71	PPV 0.80	NPV 0.86	ACC 0.82	PPV 0.61	NPV N/A	ACC 0.61	PPV 0.61	NPV N/A	ACC 0.61

*Note:* ACC = accuracy. PPV, positive predictive value (also known as precision); TRP, true positive rate (also known as recall or sensitivity).

Abbreviations: FPR, false‐positive rate; NPV, negative predictive value.

Finally, frontline physicians not specifically trained to distinguish eyelid lesions achieved a combined classification accuracy of 61%. Sensitivity toward mass detection was 65% (Table 4). Fisher’s exact test showed that VGG‐16 with threshold of 0.7 for chalazion (or 0.3 for mass) had a significantly improved performance (*p* = 0.0005) compared to the human graders. The model also showed better performance than the oculoplastic physician, although there was no statistical difference (Supporting Table 3).

**TABLE 4:** Performance of independent physicians.

	**Emergency 1**			**Emergency 2**			**Emergency 3**		
	**Mass**	**Chal.**		**Mass**	**Chal.**		**Mass**	**Chal.**	

True mass	22	22	TPR 0.5	30	14	TPR 0.68	25	19	TPR 0.57
True chalazion	10	18	FPR 0.36	10	18	FPR 0.36	13	15	FPR 0.46
	PPV 0.69	NPV 0.45	ACC 0.56	PPV 0.75	NPV 0.56	ACC 0.67	PPV 0.66	NPV 0.44	ACC 0.56

	**Optometry**			**Internal medicine**			**Combined**		
	**Mass**	**Chal.**		**Mass**	**Chal.**		**Mass**	**Chal.**	

True mass	33	11	TPR 0.75	32	12	TPR 0.73	142	78	TPR 0.65
True chalazion	17	11	FPR 0.61	14	14	FPR 0.50	64	76	FPR 0.46
	PPV 0.66	NPV 0.50	ACC 0.61	PPV 0.70	NPV 0.54	ACC 0.64	PPV 0.69	NPV 0.49	ACC 0.61

*Note:* ACC = accuracy. PPV, positive predictive value (also known as precision); TRP, true positive rate (also known as recall or sensitivity).

Abbreviations: FPR, false‐positive rate; NPV, negative predictive value.

## 4. Discussion

The primary objective of this study was to investigate the performance of deep learning models in distinguishing chalazia from eyelid masses using a heterogeneous set of photographic images from real clinical encounters taken with smartphones and other cameras typically available in an outpatient clinic or emergency/urgent care setting. The ability to function with heterogeneity in lighting, capture devise, and eye/lid positions is important for generalizable utility of such model in frontline settings where clinical environments, technology availabilities, and provider competencies can vary greatly across the world. Importantly, the authors also wish to demonstrate the effective use of this technology in a heterogeneous population with varied periocular skin complexions. It has been demonstrated that deep learning models have the ability to achieve accurate assessment of diverse skin types (i.e., Fitzpatrick skin types I‐VI) [10]. This is the first paper to the best of the authors’ knowledge demonstrating the ability of deep learning models to identify eyelid lesions in the setting of diverse skin types. The results demonstrate strong performance of deep learning algorithms in correctly identifying chalazia and eyelid masses when compared to human graders, with the VGG models performing significantly better compared to the other architectures. The adapted VGG‐16 model achieved an AUC of 0.797, compared with 0.703, 0.468, 0.454, and 0.425 for the VGG‐19, ResNet50, MobileNetV2, and Xception architectures, respectively. Though not considerably high, the AUCs achieved in our training serve as a proof‐of‐concept for the ability of AI to distinguish benign chalazion from potentially malignant masses that may require further evaluation and management by oculoplastic specialists using a heterogeneous real‐world dataset.

Specifically, our results indicated that the VGG architectures (both 16 and 19) performed well compared to the other architectures. Grad‐CAM analysis showed that the VGG‐16 model was able to focus on areas within or around the eyelid lesion for its decision‐making, indicating that the model is likely using features of the lesion itself or the contrast between the lesion and the surrounding healthy skin (lesion margin) to distinguish between mass and chalazion (Supporting Figure 1). Although there were areas of seemingly nonspecific identifications on Grad‐CAM analysis, it is possible that those areas were in fact salient features for the CNN model. While it is beyond the scope of this study to correlate specific model architecture to performance, VGG architectures with larger number of parameters (100 + M) may be more advantageous in distinguishing photographs of chalazion and mass compared to other deeper architectures with less parameters, such as Xception, ResNet50, and MobileNetV2. We speculate that the complexity of our photographs that included both the lesions in question in addition to the entire adnexal structures of various skin colors may have contributed to this observation. Given the scope of possible unique features presented in the heterogeneous image set, the architectures with larger numbers of parameters may have been advantageous in its ability to incorporate more features compared to those that are deeper but with less parameters.

Consistent with its AUC, which is the mathematical indicator of model performance, the VGG‐16 model also achieved the highest prediction accuracy of 76% as the more tangible measure of performance. While higher prediction accuracy is to be desired, adjusting prediction threshold demonstrated early promise of the AI model. When the prediction threshold for chalazion is increased to 0.6 and 0.7, VGG‐16 and VGG‐19 models showed improved or stable performances, reaching maximum accuracy of 82% and 76%, respectively. More importantly, increasing the prediction threshold of the models for chalazion significantly reduced false‐negative rate of mass prediction, yielding false‐negative rates of only 7% and 2% for VGG‐16 and VGG‐19 models, respectively. While this improvement in mass detection is accompanied by a similar increase in false‐positive rates (i.e., labeling a chalazion as a mass), the increased sensitivity to a mass does demonstrate the usefulness of the models as a clinical screening tool to detect eyelid masses that would need further referral. Additionally, given that the detection threshold is a flexible parameter, it can be adjusted based on the clinical scenario to improve clinical decision‐making. For example, threshold for mass detection may be adjusted according to age and acuity, utilizing a high mass detection threshold for young patients with acute eyelid masses and a low mass detection threshold for older patients with chronic eyelid masses.

Previous AI studies focused on eyelid lesions showed similarly promising results by accurately distinguishing benign mass from malignant mass [8, 9]. However, their general applicability to a more heterogeneous population was limited due to the uniformity of skin color included in the studies. The inclusion of skin color diversity is a significant feature of our study, as this provides representation of the wider population, as color, texture, and other high‐level features may vary significantly between different masses across various races. The difference in the proportions of eyelid lesion types among races was not statistically significant, indicating that skin color was not a factor used by our model for prediction. Additionally, our dataset included photographs of various angles, lightings, capture devices, and positions from real clinical encounters where the provider would obtain the photographs using whatever mobile device available to them. While the lack of control over the above factors (which is inherent to retrospective studies) would likely limit model performance due to the increased number of factors accounted for and does not allow for characterization of how each factor may contribute to performance, it does demonstrate the generalizability of the model to function as a first‐line screening tool using heterogeneous real‐world data. Such freedom may also open‐up possibilities of self‐screening by patients through the upload of photographs from their own mobile devices in the future to further reduce the burdens on the healthcare system.

Although our study provides valuable proof of concept for a role of AI in the early evaluation of eyelid lesions, it is important to note that this study does not intend for AI to replace the expertise of oculoplastic specialists who are proficient in discerning chalazia from eyelid masses. Its clinical utility is tailored toward a screening tool for emergency providers, primary care physicians, optometrists, and even general ophthalmologists to utilize when deciding whether a referral to an oculoplastic specialist is necessary. Such AI tools have the potential to expand the extent of clinical screening particularly in geographic regions where there is a lack of access to oculoplastic specialists (or ophthalmologists in general). For example, the tool’s improved ability to identify eyelid masses will likely lead to more prompt referral to oculoplastic specialists, reducing the chance of missing dangerous entities (e.g., sebaceous gland carcinoma) [11]. However, accurate identification of chalazia would allow for a reduced in‐office burden for the oculoplastic specialist. Finally, as briefly discussed above, the utility of this model could expand beyond clinical settings, allowing for a patient‐centric screening or telemedicine process where patient can immediately receive AI‐based feedback from their phone to ease anxiety.

This study was limited by its small sample size from a single institution. Given that the generalizability and robustness of the model largely rely on the size of the training set, a larger sample size, especially from multiple institutions across multiple geographic areas, would likely lead to more accurate and generalizable models. Due to the nature of its retrospective design, the sample size of chalazion photographs in particular was low relative to its prevalence, as most patients who presented with a chalazion did not routinely require external photographs during their visit. This may have also caused the chalazion cohort to skew toward more ambiguous cases. However, it is unclear whether the inclusion of routine and more straightforward cases would improve model performance, as what is easy for human graders may not always be easy for machines. While multiple skin colors were included in the study and the model achieved similar performance for all subgroups, the variation is by no means exhaustive, and both groups were still predominantly white. Additionally, malignant lesions were only a small percentage of the overall eyelid mass group (65/517, 13%), leaving no possibility for modeling the differences between benign and malignant lesions in this study. Larger datasets that allow for such distinction will further improve the value of such tool for frontline providers and may even find utility during encounters with oculoplastic specialists. Finally, the modeling used a dataset with known lesions, so future expansion of the model to handle cases without a lesion would further expand its utility in emergency or primary care settings. Despite its limitation, it is plausible that the efficacy of this tool would be further enhanced beyond what is reported here when deployed as a part of a clinical encounter—as the current study evaluates the tool in a vacuum, devoid of clinical history, physical examination, etc. Therefore, the utility of such model is best viewed as an additional piece of information in assisting provider decisions, rather than a replacement for clinical judgment where diagnosis is made solely based on a photograph and a model.

This study overall demonstrates the potential of AI to aid in the evaluation of eyelid lesions, particularly its possible application as a screening tool for other healthcare providers. The development of practical tools that may assist healthcare providers in characterizing eyelid lesions may be particularly helpful in settings with limited access to subspecialized care. Future investigation and collaboration are needed to continue to expand a broader dataset that would include a more comprehensive range of eyelid lesions and skin color variations, which could expand the utility of the model into oculoplastic clinics to guide biopsy decisions. By providing an additional element of assistance to healthcare providers in the assessment of eyelid lesions, deep learning algorithms have the potential to contribute to the timely and accurate identification of malignant eyelid lesions in order to improve patient outcomes and the delivery of health care.

## Author Contributions

Anfei Li designed the study, collected data, analyzed data, and constructed/reviewed the manuscript. Gabriella Schmuter constructed/reviewed the manuscript. Shehroz Rana analyzed data and constructed/reviewed the manuscript. Sayyada Hyder constructed/reviewed the manuscript. Pratik Patel, Amy Hembree, Simone Prather, and Amylisa Philips collected data. Mark Curato collected data and constructed/reviewed the manuscript. Kira Segal, Kyle J. Godfrey, and Gary Lelli designed the study, collected data, and constructed/reviewed the manuscript.

## Funding

No funding was received for this work.

## Ethics Statement

Data collection and study design were approved by the Weill Cornell Medicine Institutional Review Board (IRB record number 22—04024740). No informed consent was required for retrospective review, exempted by the Weill Cornell Medicine Institutional Review Board.

## Conflicts of Interest

The authors declare no conflicts of interest.

## Supporting Information

Supporting Table 1: ICD codes identified based on H00.∗ search and reviewed during the study.

Supporting Table 2: Diagnosis included in the current study.

Supporting Table 3: Performance of oculoplastics fellow.

Supporting Figure 1. Grad‐CAM visualization of features used for decision‐making by the VGG‐16 model. The left column illustrates the original photographs, and the right column shows features used for decision‐making as heat maps superimposed on the original photographs.

## Supporting information


**Supporting Information** Additional supporting information can be found online in the Supporting Information section.

## Data Availability

Data are available for review through the corresponding author upon reasonable request.
