# Supplementary material for: The Use of Deep Learning in Distinguishing Chalazion and Eyelid Mass
Source: J Ophthalmol. 2026 Apr 17;2026:8878251. doi: 10.1155/joph/8878251 (PMC13089635; doi:10.1155/joph/8878251)
Supplement: Supplementary file 1 — Supporting Information Additional supporting information can be found online in the Supporting Information section. [file JOPH-2026-8878251-s001.docx]

| **Supplemental Table 1: ICD codes identified based on H00.* search and reviewed during the study** | |
| --- | --- |
| **ICD 10** | **Diagnosis** |
| H00.0 | Hordeolum of lid |
| H00.01 | Hordeolum externum |
| H00.011 | Right upper lid |
| H00.012 | Right lower lid |
| H00.013 | Right unspecified lid |
| H00.014 | Left upper lid |
| H00.015 | Left lower lid |
| H00.016 | Left unspecified lid |
| H00.019 | Unspecified eye and lid |
| H00.02 | Hordeolum internum |
| H00.021 | Right upper lid |
| H00.022 | Right lower lid |
| H00.023 | Right unspecified lid |
| H00.024 | Left upper lid |
| H00.025 | Left lower lid |
| H00.026 | Left unspecified lid |
| H00.029 | Unspecified eye and lid |
| H00.03 | Abscess of eyelid |
| H00.031 | Right upper lid |
| H00.032 | Right lower lid |
| H00.033 | Right unspecified lid |
| H00.034 | Left upper lid |
| H00.035 | Left lower lid |
| H00.036 | Left unspecified lid |
| H00.039 | Unspecified eye and lid |
| H00.1 | Chalazion |
| H00.11 | Right upper lid |
| H00.12 | Right lower lid |
| H00.13 | Right unspecified lid |
| H00.14 | Left upper lid |
| H00.15 | Left lower lid |
| H00.16 | Left unspecified lid |
| H00.19 | Unspecified eye and lid |

| **Supplemental Table 2: Pathologies included in the study** | |
| --- | --- |
| **Chalazion** | **Mass** |
| Chalazion/Hordeolum  – Tissue diagnosis | Actinic Keratosis  – Tissue diagnosis |
| Chalazion/Hordeolum  – Clinical diagnosis | Basal Cell Carcinoma  – Tissue diagnosis |
|  | Dermoid Cyst  – Tissue diagnosis |
|  | Ephelis  – Tissue diagnosis |
|  | Hydrocystoma  – Tissue diagnosis |
|  | Incursion Cyst  – Tissue diagnosis |
|  | Keratoacanthoma  – Tissue diagnosis |
|  | Lentigo  – Tissue diagnosis |
|  | Lentigo Maligna  – Tissue diagnosis |
|  | Melanoma  – Tissue diagnosis |
|  | Merkel Cell Carcinoma  – Tissue diagnosis |
|  | Sebaceous Gland Adenoma  – Tissue diagnosis |
|  | Sebaceous Gland Carcinoma  – Tissue diagnosis |
|  | Seborrheic Keratosis  – Tissue diagnosis |
|  | Squamous Cell Carcinoma  – Tissue diagnosis |
|  | Squamous Papilloma  – Tissue diagnosis |
|  | Verruca Vulgaris  – Tissue diagnosis |

| **Supplemental table 3: Performance of Oculoplastics Fellow** | | | |  |
| --- | --- | --- | --- | --- |
|  | **Oculoplastics Fellow** | | |  |
|  | Mass | Chal. |  |  |
| **True**  **Mass** | 34 | 10 | **TPR**  0.77 |  |
| **True**  **Chalazion** | 10 | 18 | **FPR**  0.36 |  |
|  | **PPV**  0.77 | **NPV**  0.64 | **ACC**  0.72 |  |
| *ACC = Accuracy, TRP = True Positive Rate (also known as recall or sensitivity), FPR = False Positive Rate, PPV = Positive Predictive Value (also known as precision), NPV = Negative Predictive Value | | | |  |

**Supplemental Figure 1.**


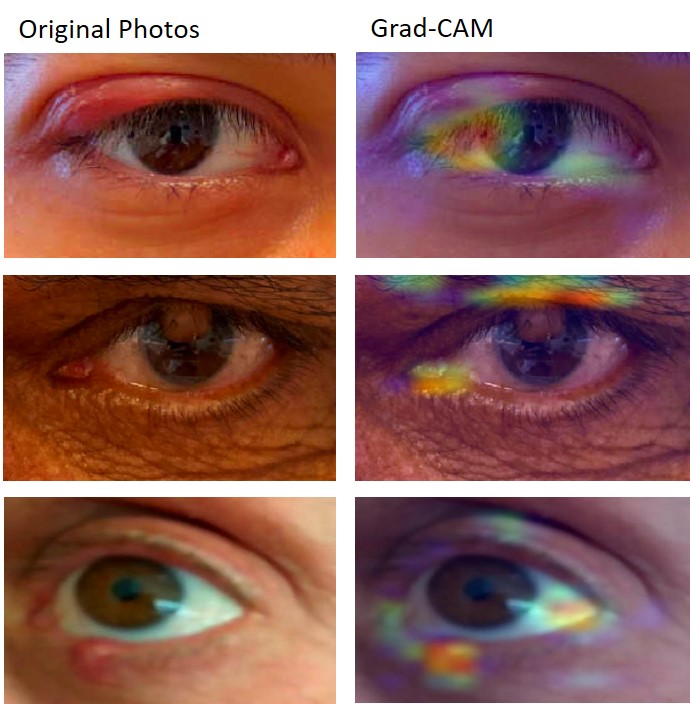


**Supplemental Figure 1.** Grad-CAM visualization of features used for decision-making by the VGG-16 model. The left column illustrates the original photos, and the right column shows features used for decision-making as heat maps superimposed on the original photos.
